# Visual Detection of Road Cracks for Autonomous Vehicles Based on Deep Learning

**DOI:** 10.3390/s24051647

**Published:** 2024-03-03

**Authors:** Ibrahim Meftah, Junping Hu, Mohammed A. Asham, Asma Meftah, Li Zhen, Ruihuan Wu

**Affiliations:** 1 College of Mechanical and Electrical Engineering, Central South University, Changsha 410017, China; ibrahim4mch@csu.edu.cn (I.M.); 213711021@csu.edu.cn (L.Z.); ruihuanwu@csu.edu.cn (R.W.); 2 School of Computer Science and Engineering, Central South University, Changsha 410017, China; m.asham@csu.edu.cn (M.A.A.); asma4it@csu.edu.cn (A.M.)

**Keywords:** transfer learning, Random Forest, road information, autonomous vehicle

## Abstract

Detecting road cracks is essential for inspecting and assessing the integrity of concrete pavement structures. Traditional image-based methods often require complex preprocessing to extract crack features, making them challenging when dealing with noisy concrete surfaces in diverse real-world scenarios, such as autonomous vehicle road detection. This study introduces an image-based crack detection approach that combines a Random Forest machine learning classifier with a deep convolutional neural network (CNN) to address these challenges. Three state-of-the-art models, namely MobileNet, InceptionV3, and Xception, were employed and trained using a dataset of 30,000 images to build an effective CNN. A systematic comparison of validation accuracy across various base learning rates identified a base learning rate of 0.001 as optimal, achieving a maximum validation accuracy of 99.97%. This optimal learning rate was then applied in the subsequent testing phase. The robustness and flexibility of the trained models were evaluated using 6,000 test photos, each with a resolution of 224 × 224 pixels, which were not part of the training or validation sets. The outstanding results, boasting a remarkable 99.95% accuracy, 99.95% precision, 99.94% recall, and a matching 99.94% F1 Score, unequivocally affirm the efficacy of the proposed technique in precisely identifying road fractures in photographs taken on real concrete surfaces.

## 1. Introduction

Navigating autonomous vehicles reliably on roadways demands robust crack detection and path planning algorithms. Autonomous vehicles are rapidly gaining traction across diverse domains, including material transport, environmental exploration, and agricultural tasks like harvesting, and weeding on roads and crops. Current autonomous vehicle navigation models usually emphasize steering clear of obstacles, neglecting the influence of road cracks. Over time, highways incur the formation of cracks that can present substantial obstacles for autonomous vehicles. Significant cracks might hinder the development of autonomous vehicles, while minor cracks can lead to deviations from predetermined routes, thereby enhancing the probability of accidents. In order to overcome these challenges, it is essential that autonomous vehicle navigation systems integrate crack detection and protection systems. Drawing inspiration from nature’s remarkable abilities, biomimetic AI can empower autonomous vehicles to navigate complex environments, including roads riddled with cracks. By dynamically adjusting running speeds, vehicles can safely traverse these cracks, ensuring both efficiency and safety. This biomimetic approach paves the way for smarter, safer roads, revolutionizing autonomous navigation. Sensors in connected and autonomous vehicles (CAVs) collect tremendous amounts of data. Experts are figuring out how to use this data to improve CAVs. Machine learning is an effective tool that has already achieved a great deal with this data, and it will certainly have a significant role in the future development of CAVs. Friction from vehicles and the weather combine to cause pavement cracks. Overlooking them may lead to accidents and costly repairs. Early crack detection is crucial, but conventional manual approaches are expensive and time-consuming. Computer vision technologies are being used by researchers to create automatic ways to find cracks by introducing intelligent automation systems. While the first models were simple, the more complex versions use techniques like temporal image comparison, wavelet features, and local binary patterns to track the development of cracks. This is just the initial stage of the endeavor, where AI will play an essential part in building more secure and economical roads.

Some researchers have delved into traditional computer vision (CV) algorithms that use image geometry for wide-ranging crack detection, which are proficient at operating in environments with noise, poor lighting, and shadows. However, these techniques face challenges in precisely identifying complex crack patterns or handling uneven intensity. In contrast, model-based methods excel by utilizing the essential structure of cracks for more effective detection, especially useful in difficult conditions. These methods are similar to using a design for identifying cracks, enabling them to identify tiny cracks that might be overlooked by conventional approaches in shadowy or noisy settings. Despite their advantages, model-based techniques also have their limitations, particularly when challenged with intricate crack patterns or varying intensities. While they are more capable of handling noise and consistently detecting cracks, these specific conditions can still pose a challenge.

The significance of a crack [1,2] is assessed based on its dimensions, extent, and placement, thereby demanding the continuous observation of the structural stability and functionality to ensure the durability of any infrastructure. Conventional crack detection methods mainly rely on visual inspections, which are time-consuming and demand substantial manual effort. The effectiveness of these inspections primarily relies on the inspector’s proficiency, knowledge, and perception. However, these labor-intensive techniques frequently consume a significant amount of time and may result in the delayed detection of the most important cracks that are approaching disastrous conditions. In order to address these limitations, recent progress has been made in improving the detection process through the shift from manual to automated by introducing more advanced methods. Automation is increasingly relying on deep learning (DL) [3,4] and computer vision techniques, particularly image processing (IP) and aerial imagery. Automated crack detection using these images minimizes costs and time for inspections while also improving the safety and reliability of inspections for concrete constructions.

Concrete cracking is a complex phenomenon that is affected by several components, including the rate at which it dries, its tensile strength, elasticity, and the level of pressure it experiences. The presence of excessive water in concrete causes it to shrink and crack during the drying process, resulting in the formation of different types of cracks such as plastic shrinkage, map, and diagonal corner cracking. While the majority of damage is not considered a threat to the structural integrity, there are circumstances where severe cracks are able to impair it. Researchers have been primarily investigating automatic crack identification in concrete by employing image-based techniques such as thresholding and morphological methodologies, in addition to machine learning approaches like convolutional neural networks (CNNs) [5,6,7,8,9,10]. Despite this innovation, there exists a deficiency in grasping the impact of poor images on the detection of cracks, as well as the extent to which image preprocessing can alleviate these concerns. As a result of this gap, there has been research conducted to investigate the influence of image preprocessing on the efficacy of pretrained CNN models in detecting cracks. Below are the main contributions of this paper.

The main challenge [11,12,13,14,15] in detecting road cracks arises from the diverse types of cracks caused by severe weather and long-term vehicle use. Traditional manual detection methods are inefficient and often miss cracks, while machine learning (ML) [16,17,18,19,20] models require manually designed features for crack detection. According to the Federal Highway Administration (FHWA), there are various crack types like fatigue, longitudinal, and transverse, making it difficult to design a universal feature extraction model for effective automatic detection using machine learning. This limitation has led to the adoption of deep learning (DL) in road crack detection for autonomous vehicles. Deep learning [21,22,23,24], utilizing sophisticated neural networks such as CNNs, addresses these challenges by processing extensive visual data from vehicle-mounted cameras. Trained on large datasets, these models learn to discern intricate patterns and discrepancies indicative of cracks, enabling real-time detection and analysis of road surface anomalies. This approach not only enhances the decision-making capabilities of autonomous vehicles but also ensures timely reactions to road conditions and upholds safety standards. The application of deep learning thus significantly improves the vehicles and proficiency in handling varied and complex road surfaces.

Pavement cracking is a common and major concern, affecting pavement serviceability. Understanding these cracks helps engineers assess conditions and decide on the right maintenance or rehabilitation strategies. A thorough understanding of pavement cracking is key for engineers to accurately assess pavement conditions and choose effective maintenance strategies. This knowledge leads to timely repairs, preventing further damage, and extends the lifespan of road infrastructure. The study investigated the impact of pavement structure and surface conditions on deflection measurements using traffic speed deflection devices. Through non-parametric rank correlation and Random Forest regression analyses, it was found that pavement type, surface roughness, and distress significantly influence deflection. The study also discovered a strong link between surface condition indices and deflection, especially on flexible pavements.

- The paper addresses the critical task of road crack detection, which is vital for inspecting and assessing concrete pavement structures’ integrity, particularly in scenarios like autonomous vehicle road detection.
- Traditional image-based methods often require complex preprocessing to extract crack features, making them less practical for real-world scenarios where concrete surfaces exhibit various forms of noise. This research provides a solution that mitigates these preprocessing challenges.
- The paper presents an innovative approach that combines a Random Forest machine learning classifier with a DCNN. This hybrid approach leverages the strengths of both techniques to enhance crack detection performance.
- The paper identifies an optimal base learning rate of 0.001 through rigorous experimentation, achieving an impressive maximum validation accuracy of 99.97%. This rate is crucial for model training and contributes to the overall effectiveness of the approach.

In the subsequent sections, we will begin by providing a concise overview of previous efforts in road crack detection in Section 2. Following this, Section 3 will elaborate on the specifics of our proposed methodology. Section 4 will be dedicated to discussing the implementation details and performance evaluation of our model. In the forthcoming sections, we will delineate the discussion, limitations, and strengths of our approach, and a conclusion for this study.

## 2. Related Work

Autonomous vehicles [25,26,27,28,29,30], while able to navigate over small road cracks, risk significant path deviations during high-speed maneuvers, potentially causing collisions. It is crucial for these vehicles to dynamically adjust speeds, such as decelerating near cracks, for safety and precision. Concurrently, the advancement of computer science, especially deep learning, has significantly improved road crack detection, a vital aspect of road maintenance. This technological progress facilitates automated feature analysis, surpassing traditional, error-prone methods like manual inspection. However, most research [31,32,33,34,35,36,37,38,39,40] has focused more on crack detection and classification, often neglecting the detailed analysis and measurement of specific crack characteristics [41,42,43,44]. Study [45] used laser-scanned range images to classify roadway cracks with a deep convolutional neural network (DCNN). They evaluated 36 DCNN architectures and found that a seven-layer DCNN with constant 7 × 7 kernels and increasing network widths yielded the highest classification performance. The proposed approach detected cracks consistently and accurately under diverse situations, although hyperparameter adjustment is still needed for maximum performance.

Over the past few decades, several thoroughly studied techniques have been developed to address the challenges in navigation and motion planning. These encompass methods such as learning-based models, neural network models, sampling-based algorithms, and evolutionary computational models, among various others. In [13], improved the sampling-based rapidly exploring random tree (RRT) algorithm in 2021. This improved version considers mobile robots’ considering their kinodynamic constraints to produce a collision-free track. However, sampling-based algorithms usually provide stochastic consistency and inefficient pathways. Author [34] highlight the exceptional efficacy of vibration-based methods for detecting and classifying road surface anomalies. Nonetheless, they acknowledge significant challenges in the reproducibility and consistency of these methods. These issues primarily arise from limited and complex challenging scenarios, small sample sizes, and the unavailability of datasets for public use, contributing to the difficulties encountered in this field. Developed [24] a hybrid fireworks algorithm utilizing LIDAR-based local navigation, proficient at producing concise, collision-free trajectories in unstructured settings. However, a notable limitation of this method is its potential inefficiency in complex or highly dynamic environments, where real-time adaptation is crucial.

A domain knowledge-based genetic algorithm (GA) for optimal collision-free trajectory generation between a source and multiple targets is introduced by [34]. This GA integrates four novel domain knowledge-based operators specifically designed for efficient path planning. However, its effectiveness may diminish in scenarios with rapidly changing environments or unpredictable obstacles due to its reliance on static domain knowledge. A hybrid approach for path planning in autonomous surface vehicles was developed by [5,6] using the ant colony optimization (ACO) and artificial potential field (APF) methods. However, the study lacks a discussion on the computational complexity of this hybrid algorithm and does not offer a comparative analysis of its efficiency against other established algorithms. Developed [23] a cost-effective unmanned aerial vehicle (UAV) equipped with sensors like LIDAR and optics for inspecting concrete pavement structure cracks [7,8]. Utilizing the SVM algorithm for data classification, this UAV overcomes the challenges of real-time crack detection [9,10,14,15,17,18,19] within budget constraints. While the method’s computational efficiency is a major advantage for low-cost UAVs, the paper does not address how this efficiency might impact the accuracy or reliability of the inspection results [29,30,31,32,33,34,35,36,37,38,39,40].

Introducing a novel lightweight MobileNetV2_DeepLabV3 [31] image segmentation network specifically tailored for crack detection addresses critical concerns regarding assessment reliability, safety, accuracy, and cost-effectiveness. This innovative approach involves fine-tuning the parameters of the DeepLabV3+ network’s atrous spatial pyramid pooling (ASPP) module to mitigate interference from common sources of noise, light variations, shadows, and other environmental factors. This optimization enables more precise segmentation of long-range objects, enhancing the overall performance and robustness of the crack detection system.

An intelligent robotic system for autonomous concrete tunnel crack identification and characterization was developed by [11]. This system provides a comprehensive solution for identifying and analyzing cracks. Its autonomous navigation and extensive tunnel integrity enhance the efficiency and thoroughness of the inspection process. However, the paper does not address any potential limitations of this system, such as constraints in operational environments or challenges in detecting certain types of cracks. Study [19] developed Panthera, a robotic platform for road crack segmentation and garbage detection. However, their study does not address the limitations of using the mobile mapping system (MMS) for geotagging defects, nor does it explore the technique’s scalability across larger or varied pavement types [40,41,42,43,44,45,46,47].

An innovative approach to automated 3D crack detection for structures is presented, leveraging the fusion of LiDAR and camera data. This method capitalizes on precise extrinsic sensor calibration to ensure the accurate registration of images and LiDAR point clouds. By integrating these technologies, the proposed system enhances the detection and analysis of cracks with unparalleled accuracy and efficiency, offering a robust solution for structural monitoring and maintenance.

## 3. Materials and Methods

### 3.1. Dataset Details

In our investigation [27,28], we harnessed a publicly accessible Kaggle dataset, comprising a vast collection of thirty thousand photographs capturing concrete and pavement surfaces. These images were categorized into two distinct groups: “negative” and “positive”. Originating from the Nigerian Army University in Biu, Borno, they were acquired through aerial imagery captured using a DJI Mavic 2 Enterprise drone and ground-level shots taken with a smartphone, representing a comprehensive perspective. To facilitate processing and analysis, the dataset was standardized to a resolution of 227 by 227 pixels and stored in the universally compatible RGB JPEG format. Table 1 details a dataset specifically for a binary classification problem. Regarding the number of data samples allocated for training the classification model, for both the “Negative” and “Positive” classes, there are 12,000 training samples each showing the number of data samples reserved for testing the classification model. Similar to the training data, there are 3000 testing samples for both the “Negative” and “Positive” classes. There are a total of 24,000 data samples for training (12,000 “Negative” + 12,000 “Positive”) and 6000 data samples for testing (3000 “Negative” + 3000 “Positive”). In total, there are 30,000 data samples in this dataset.

### 3.2. Preprocessing and Augmentation

This step involves adjusting the dimensions of all the images in dataset to a standard size of 224 × 224 pixels. Most pre-trained neural network architectures, such as those in popular deep learning libraries like TensorFlow, are designed to work with this standard input size. Data augmentation is a technique used to artificially increase the diversity of your training dataset by applying various transformations to the images. This helps improve model generalization and reduce overfitting.

- Rotation (90 degrees)

Images are rotated by 90 degrees in increments. This means we create additional training samples by turning the original images 90, 180, and 270 degrees. This helps the model become invariant to object orientation, making it better at recognizing objects from different angles.

- Vertical Flip

Images are flipped vertically along the horizontal axis. This mirrors the images vertically, creating a new set of training samples. It helps the model learn from vertically inverted images and adds diversity to the dataset.

- Horizontal Flip

Images are flipped horizontally along the vertical axis. This mirrors the images horizontally, creating another set of training samples. Like vertical flip, it introduces variations in object orientation.

**Figure 1:**
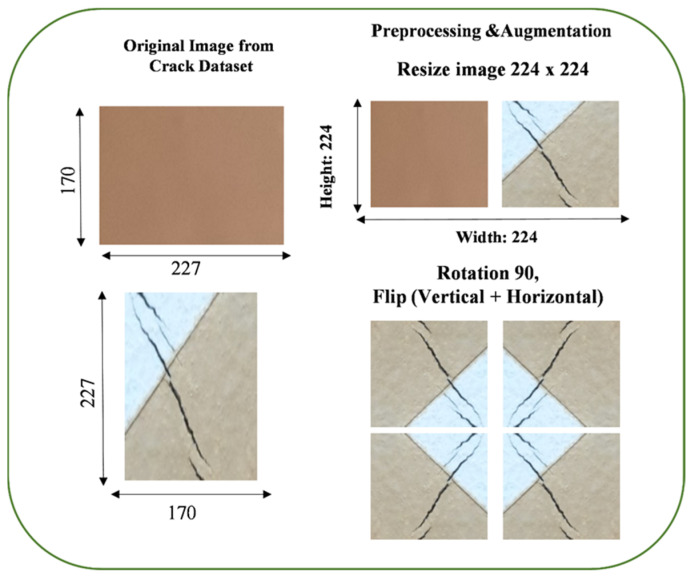
Sample images of augmented and resized images.

These augmentation techniques collectively result in an augmented dataset with a broader range of variations, making the model more robust and capable of handling diverse inputs. This helps the model generalize better during training. It reduces the risk of overfitting, where the model becomes too specialized in recognizing the training data but needs help with new, unseen data. Figure 1 displays sample images of augmented and resized images.

### 3.3. Transfer Learning

Transfer learning is a machine learning technique that revolutionizes the training of neural networks, particularly in computer vision tasks such as image classification. Unlike traditional convolutional neural networks (CNNs), where models are trained from scratch on a specific dataset, transfer learning takes a more efficient and practical approach. In traditional CNNs, the model learns all its parameters and features from the ground up, often requiring an extensive dataset and substantial computational resources. In contrast, transfer learning leverages pre-trained neural network models already trained on vast and diverse datasets, typically for general image recognition tasks. These pre-trained models have acquired a wealth of knowledge regarding feature extraction and hierarchical representation learning. Instead of starting from scratch, transfer learning fine-tunes these pre-trained models on a target dataset or task. By adapting the learned features to the new study, transfer learning reduces the labeled data and training time needed to achieve impressive results. This approach enhances model efficiency and improves performance, making it a powerful tool for various applications, especially when labeled data are limited.

#### 3.3.1. MobileNet

Developed by [11] and first presented in 2017, MobileNet is a category of efficient models designed specifically for mobile and embedded vision applications. The key feature of the architecture is the deployment of depthwise separable convolutions, a method that effectively decreases the size and computation requirements of the model. MobileNetV1’s innovative methodology enables it to achieve an optimal balance of efficiency and accuracy, making it especially well-suited for devices with constrained computational capabilities. Its optimization for speed and adaptability for deployment on various devices have made it a preferred choice for real-time applications in mobile settings. The MobileNetV1 architecture has been extensively utilized in numerous study schemes, highlighting its efficacy and adaptability in the domain of lightweight deep neural networks.

#### 3.3.2. InceptionV3

Introduced in 2015 by Google, InceptionV3 is a key development in the Inception series of convolutional neural networks and was further detailed in a 2017 publication. This model marks a significant evolution in the series, incorporating advancements like factorized convolutions, label smoothing, and auxiliary classifiers to increase lower-level learning within the network. Particularly, InceptionV3 employs multiple kernel sizes in the same layer, allowing it to capture spatial hierarchies more effectively in images. Designed primarily for complex, large-scale image recognition tasks, InceptionV3 stands out for its high accuracy. While it offers improved classification performance, it does so with consideration for computational efficiency, although with a higher computational requirement than models like MobileNetV1. The effectiveness and improvements brought by InceptionV3 have led to its widespread application in various research articles focused on image classification.

#### 3.3.3. Xception

Xception, an abbreviation for “Extreme Inception”, is a convolutional neural network architecture that builds upon and extends the concepts of InceptionV3. It was first presented in 2016 and formally introduced in 2017. Xception [3] is distinct for its use of depthwise separable convolutions. This design choice not only aligns with the Inception model’s viewpoint but also enhances the network’s depth and efficiency. As a result, Xception surpasses its prototypes in large-scale image recognition tasks by delivering superior accuracy. Although Xception requires more computational power compared to models such as MobileNetV1, it outperforms other models in situations where superior performance is essential and abundant computational resources are available, demonstrating its effectiveness in tackling complex image recognition tasks.

### 3.4. Machine Learning Classifier

Machine learning techniques [12], notably in recent studies, have enhanced the accuracy of detecting road surface cracks. Traditional approaches often overlooked varying illumination conditions affecting the target. One innovative method, IlumiCrack, combined Gaussian mixture models and CNN object detection models, like YOLO and SSD, to accurately categorize various types of road cracks under diverse lighting. Another study employed adaptive density-based fuzzy c-means clustering and hybrid wavelet–Walsh transform for effective crack detection and classification. These advancements show how deep learning and consideration of environmental factors significantly improve road damage detection. Authors developed a comprehensive machine learning model for detecting and classifying road cracks in urban settings. This model follows a process involving image segmentation, noise reduction, feature extraction using Hough transform and heuristic equations, and finally, crack classification. It employs various classification algorithms like neural networks, SVM, decision trees, KNN, bagged trees, and a novel hybrid model. The hybrid model, in particular, demonstrated enhanced accuracy, achieving an overall accuracy rate of 93.86% in experimental tests.

#### Random Forest

The Random Forest algorithm is a popular machine learning method used for both classification and regression tasks. It operates by constructing multiple decision trees during training and outputs the mode of the classes (classification) or mean prediction (regression) of the individual trees. The Random Forest algorithm, an ensemble learning method, is notably utilized in road crack detection for its accuracy in classification and regression tasks. This introduced Crack Forest, a framework using random structured forests for improved road crack detection. This method leverages integral channel features and a new crack descriptor for enhanced crack representation and noise distinction, demonstrating superior precision compared to other methods. This high level of precision underscores the model’s efficiency and potential for application in various research areas. In the field of road crack detection and classification, the Random Forest algorithm is highly valued for its effectiveness. This approach is particularly advantageous in handling the complexities of road surface images, where it aids in accurately identifying cracks. The algorithm’s ability to manage various data features and its robustness against overfitting make it a preferred choice for classifying and detecting road cracks in diverse and challenging environments.

### 3.5. Ensemble Learning

Ensemble learning is a machine learning technique that combines multiple models to improve the accuracy and robustness of the predictions. Ensemble learning in crack detection utilizes multiple learning algorithms or models to enhance the accuracy and robustness of identifying cracks, a crucial aspect in structural health monitoring and civil engineering. This approach is particularly beneficial because different models have varying strengths in detecting cracks under diverse conditions, thus improving overall performance. Ensemble methods are more robust against noise and variability in crack appearances, ensuring better generalization to unseen data by balancing individual model biases. They are particularly adept at handling complex crack patterns, reducing the variance of predictions, and offering higher adaptability to different types of cracks and conditions like lighting and background.

The key benefits of employing ensemble learning in road crack detection include a significant increase in detection accuracy, a reduction in false positives and negatives, and enhanced scalability and flexibility. By aggregating predictions from multiple models, ensemble methods effectively balance weaknesses of individual models, leading to more stable and reliable performance. This adaptability and scalability make ensemble learning an invaluable tool in ensuring structural integrity and safety across various engineering applications, where accurate crack detection is paramount.

### 3.6. Proposed Architecture

Our methodology begins with thorough data preparation, involving collecting and dividing a diverse set of labeled road images into training, validation, and testing subsets. In cases where computational resources are limited, or the available training data need enhancement, TL becomes invaluable. TL empowers us to leverage models pre-trained on extensive benchmark datasets, thereby elevating classification accuracy when dealing with smaller datasets. Recent years have witnessed TL’s effectiveness and promise in various imaging applications. What sets our work apart is its application of TL in the context of road information categorization (crack vs. non-crack) for autonomous vehicles. Utilizing TL in crack image classification enables us to harness patterns and insights derived from extensive training on diverse benchmark datasets, ensuring precise and reliable results even with limited data.

Our research focuses on three pivotal pre-trained CNN models, which constitute the core of our investigation. MobileNet, a CNN architecture tailored for efficient on-device image classification tasks, stands out for its exceptional efficiency achieved by implementing depthwise separable convolutions. These convolutions significantly reduce computational costs while maintaining high accuracy. MobileNet’s widespread adoption in mobile and embedded applications makes it an ideal choice for real-time image recognition on resource-constrained devices. The Xception model, another prominent CNN architecture, excels in image classification tasks. It attains remarkable accuracy by utilizing depthwise separable convolutions, effectively reducing parameter counts and computational complexity. This design does not compromise its ability to capture intricate image features, making Xception a popular choice for computer vision applications such as image recognition and object detection. InceptionV3, our third focal model, is a deep CNN architecture renowned for its image classification and feature extraction capabilities. Its intricate network of inception modules empowers it to capture features across various scales, enabling the acquisition of both global and local image features. InceptionV3 has gained widespread recognition in computer vision due to its impressive performance and efficient architectural design. Utilizing the capabilities of three pre-trained models, we can effectively use their existing features to classify images of roads into crack and non-crack categories. These pre-trained models prove invaluable in discerning road anomalies and patterns relevant to autonomous vehicles, as they have already acquired the ability to recognize common patterns and characteristics across a wide range of image categories.

To significantly enhance the precision of crack and non-crack road detection for autonomous vehicles, we harness the capabilities of three pre-trained models: Xception, MobileNet, and InceptionV3. Instead of relying on their final classification layers, we opt for feature extraction from intermediate layers, which capture intricate and distinctive information. The crux of our approach lies in the stacking phase, where we consolidate the feature vectors obtained from Xception, MobileNet, and InceptionV3 to construct a comprehensive ensemble representation for each image. This fusion of diverse features enables our model to leverage the unique strengths of each architecture, ultimately augmenting the overall discriminative power of the system.

**Figure 2:**
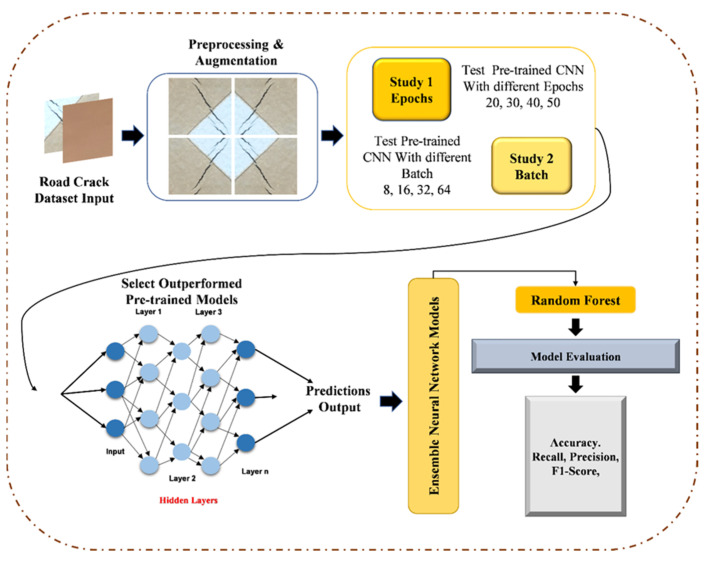
Overview of proposed architecture diagram.

In our pursuit of optimal performance, we explore various machine learning techniques utilizing these stacked features to classify road data (crack vs. non-crack) for autonomous vehicles. Subsequently, we train a classifier, such as a Random Forest, on the stacked feature representations using the training dataset, with the validation set aiding in fine-tuning hyperparameters to achieve peak efficiency. The evaluation phase is paramount, where we meticulously assess the proposed model’s accuracy, precision, recall, F1 Score, and other pertinent metrics on the test dataset. We experiment with different stacking techniques for further performance enhancements and delve into ensemble learning approaches like bagging, boosting, Min Ensemble, Max Ensemble, and Average Ensemble. Employing cross-validation ensures the model’s robustness and its capacity for generalization. Upon achieving the desired accuracy, our model is poised for real-world deployment in road maintenance and safety monitoring applications. By effectively harnessing the combined capabilities of Xception, MobileNet, InceptionV3, and Random Forest, we deliver a potent solution for accurate crack detection in road infrastructure. Figure 2 presents overview of proposed architecture diagram.

## 4. Results and Implementation

The proposed model, which leverages the stacked features from Xception, MobileNet, and InceptionV3 along the Random Forest classifier, undergoes a rigorous evaluation to ensure its effectiveness in crack and non-crack road detection. Following the model development, the evaluation phase includes several crucial steps. This section describes the experimental setup, model evaluation, proposed model hyperparameter details, and class wise proposed model performance.

### 4.1. Experimental Setup

The methods and results of the experiments on the suggested architectures are described in depth in this section. This study contrasted the ensemble method suggested for the competitive model with deep transfer learning using the ML classifier. We assess the performance of the proposed technique using the road crack public dataset. Accurate crack classification is the main goal of this endeavor. Python and the Keras package were utilized to train the suggested model. The GPU runtime type on the Google Collaboratory Notebook is used for any experimental research. This is a free academic study program from Google. The model was trained using 4 GB of RAM and 12 GB of memory on the NVIDIA GeForce MX350 (Nvidia, Santa Clara, CA, USA).

### 4.2. Model Evaluation

The evaluation begins by subjecting the model to a dedicated test dataset, distinct from the training and validation sets. This separation ensures an unbiased assessment of the model’s performance. Various performance metrics are then meticulously computed to evaluate its classification capabilities comprehensively. These metrics include the following:

- Accuracy

This metric gauges the overall correctness of the model’s predictions, measuring the proportion of correctly classified samples.

- Precision

Precision assesses the model’s ability to make accurate positive predictions. It quantifies the ratio of true positive predictions to the total number of positive predictions made by the model.

- Recall

Recall, also known as sensitivity or true positive rate, measures the model’s effectiveness in identifying all relevant positive class instances. It calculates the ratio of true positives to the total number of actual positive instances.

- F1 Score

The F1 Score is a harmonized metric that balances precision and recall. It considers false positives and false negatives, providing a single value that summarizes the model’s overall performance in binary classification tasks.

- Confusion Matrices

Confusion matrices are essential visual tools that showcase the model’s classification results in a tabular format. They break down predictions into true positives, false positives, and false negatives, allowing for in-depth analysis of classification errors.

By meticulously assessing the model’s performance using these metrics and interpreting the information presented in the confusion matrices, we comprehensively understand its strengths and weaknesses in the context of crack and non-crack road detection. This rigorous evaluation ensures the model’s reliability and suitability for practical applications.

(1) $$Accuracy\;(AC)=\frac{TP+TN}{TP+FP+TN+FN}$$

(2) $$Precision\;(PR)=\frac{TP}{TP+FP}$$

(3) $$Recall\;(RC)=\frac{TP}{TP+FN}$$

(4) $$F1\;Score\;(FS)=2\times \frac{Precion\times Recall}{Precision+Recall}$$

### 4.3. Hyper Parameter Details

Image Size (224 × 224): Image size refers to the dimensions of the input images used in the model. In this case, the images are resized to 224 pixels in height and 224 pixels in width. This size is a common choice for many pre-trained deep-learning models, including those used for image classification. Epochs (10): An epoch represents one complete pass through the entire training dataset during model training. In this case, the model will iterate over the whole dataset 10 times. The number of epochs is a hyperparameter that determines how often the model learns from the data. Loss Function (Binary): In this context, the loss function is set to “Binary”. This indicates that the model is likely being used for a binary classification task, such as crack vs. non-crack road detection. The binary loss function typically involves measuring the difference between the two classes’ predicted values and actual labels. Optimizer (Adam): Adam (short for Adaptive Moment Estimation) is a popular optimization algorithm that updates the model’s parameters during training. It combines elements of both the RMSprop and momentum optimization techniques to adjust learning rates for each parameter efficiently. Activation (Sigmoid): The activation function used in the output layer of the model is the sigmoid activation function. Sigmoid is often used for binary classification tasks because it maps the model’s raw output to a probability value between 0 and 1, which can be interpreted as the probability of the input belonging to the positive class. Learning Rate (0.001): The learning rate is a hyperparameter that controls the step size at which the model’s parameters are updated during training. A learning rate of 0.001 indicates that the model makes minor parameter adjustments in each training iteration. Learning rate tuning is crucial, as it affects the convergence and stability of the training process.

Batch Size (32): The batch size refers to the number of samples processed together in each training iteration. A batch size of 32 means 32 images are used in each training step before updating the model’s parameters. Batch size impacts training speed and memory usage and is another hyperparameter that needs to be tuned for optimal performance. These parameter details provide essential information about how the deep learning model is configured for training and inference. Adjusting these parameters, along with appropriate data preprocessing and model architecture, is essential for achieving the desired accuracy and performance in specific tasks like crack and non-crack road detection.

### 4.4. Classification Performance

Table 2 presents a comparative performance analysis of different models for a crack and non-crack road detection task, evaluating their precision, recall, F1 Score, and accuracy. The base models used for this analysis are MobileNet, InceptionV3, and Xception, and they are compared against a proposed model. MobileNet achieved a remarkable balance between precision, recall, F1 Score, and accuracy, scoring 98.13%. This demonstrates its ability to effectively classify crack and non-crack road segments, making it a strong candidate for the task. InceptionV3 also exhibited impressive performance across all metrics, with precision, recall, F1 Score, and accuracy measuring 96.38%. This indicates its capability to handle the detection task effectively. Xception, another base model, delivered results very close to InceptionV3, with precision, recall, and accuracy, all at 96.20% and an F1 Score of 96.19%. This suggests that it is a competitive choice for the crack detection problem. The proposed model outperformed all the base models significantly. It achieved an exceptional precision of 99.95%, recall of 99.94%, F1 Score of 99.94%, and accuracy of 99.95%. These enhanced results indicate that the proposed model has substantially improved the ability to distinguish between crack and non-crack road segments compared to the base models, making it an efficient choice for this task. The proposed model demonstrates superior precision, recall, F1 Score, and accuracy performance compared to the base models (MobileNet, InceptionV3, and Xception). It shows promise in crack and non-crack road detection, offering higher accuracy and reliability, which could be valuable in real-world applications like road maintenance and safety monitoring. Furthermore, Table 2 also presents the test time required by each base model as well as our proposed model.

**Figure 3:**
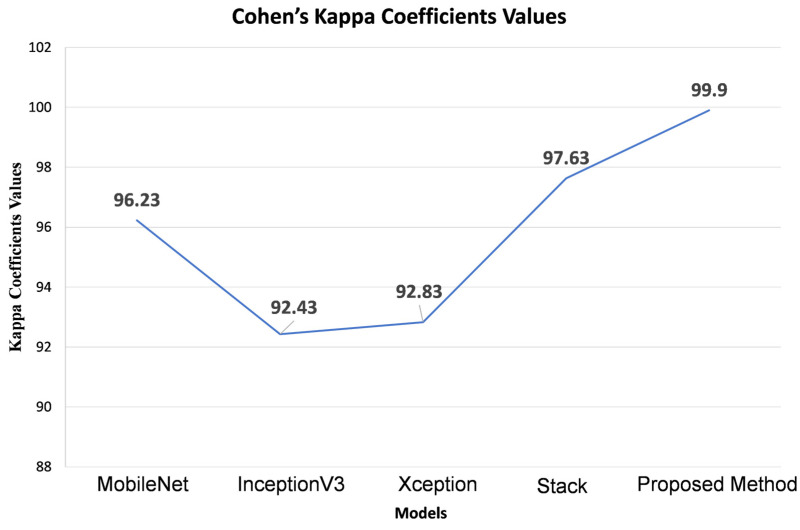
Cohen’s Kappa coefficient comparison between baselines, stack, and proposed model.

The results Figure 3 displayed in provide a comprehensive view of the Kappa coefficients and error values for various models assessed on the test dataset. Following the evaluation of base models, including MobileNet, Xception, InceptionV3, and a stacked ensemble, the respective Kappa coefficients were found to be 96.23, 92.43, 92.83, and 97.63. In contrast, our proposed model exhibited a remarkable enhancement in the Kappa value, achieving a coefficient of 99.9. Moreover, the graphical representation illustrates a consistent decrease in error values as the Kappa coefficients increase, signifying improved predictive accuracy with higher Kappa values. This substantial boost in the Kappa coefficient highlights our proposed model’s exceptional robustness and reliability in classifying road information (crack vs. non-crack) for autonomous vehicle predictions. The outcomes underscore the significant performance enhancement delivered by our suggested model, which leverages the stack ensemble and Random Forest algorithm to weigh the base models. Our proposed model outperforms the evaluated base models across various metrics, including accuracy scores, Kappa coefficients, F1 Score, and precision values. The Random Forest’s superior predictive accuracy and adaptability in handling both regression and classification tasks and its ability to provide feature relevance scores for feature selection make it a valuable asset. Our suggested model’s capability to harness the ensemble models’ complementary features to overcome individual models’ limitations results in a substantial overall performance improvement. These findings underscore the potential of our suggested model as an effective approach for autonomous vehicle crack vs. non-crack detection.

The Kappa coefficient measures the agreement between observed and expected agreement beyond chance, providing insight into the reliability of classification systems. Our base model, with Kappa coefficient values ranging from 92.43 to 97.63, showcases a solid performance, while the proposed model, boasting a Kappa coefficient of 99.9, demonstrates remarkable improvement in agreement metrics, indicating superior classification accuracy.

**Figure 4:**
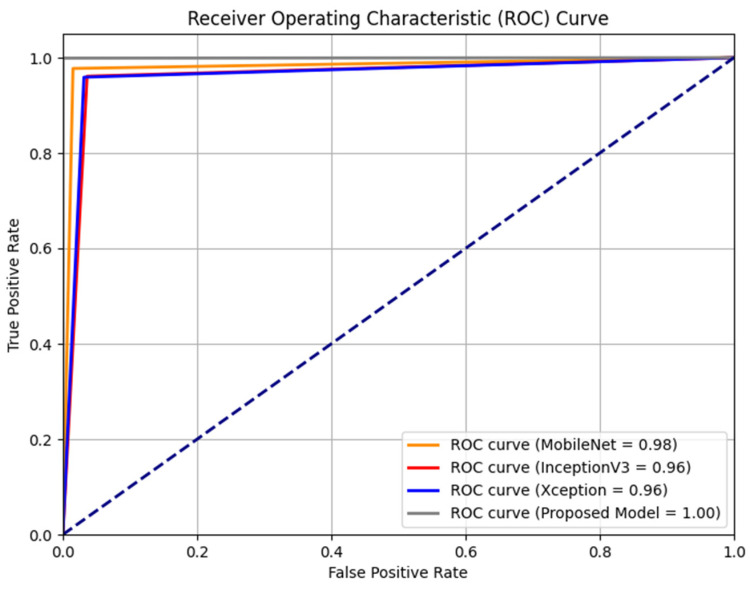
Overview of ROC curve between proposed model and baselines.

The performance of each base model was assessed by evaluating their sensitivity and specificity rates through the ROC curve, as depicted in Figure 4. The results indicated that the MobileNet, InceptionV3, and Xception baseline models outperformed the original baseline models, achieving AUROCs of 0.98, 0.96, and 0.96, respectively. Our models’ remarkable performance can be attributed to the synergistic utilization of multiple models, which enhances their overall strength compared to the baseline models.

**Figure 5:**
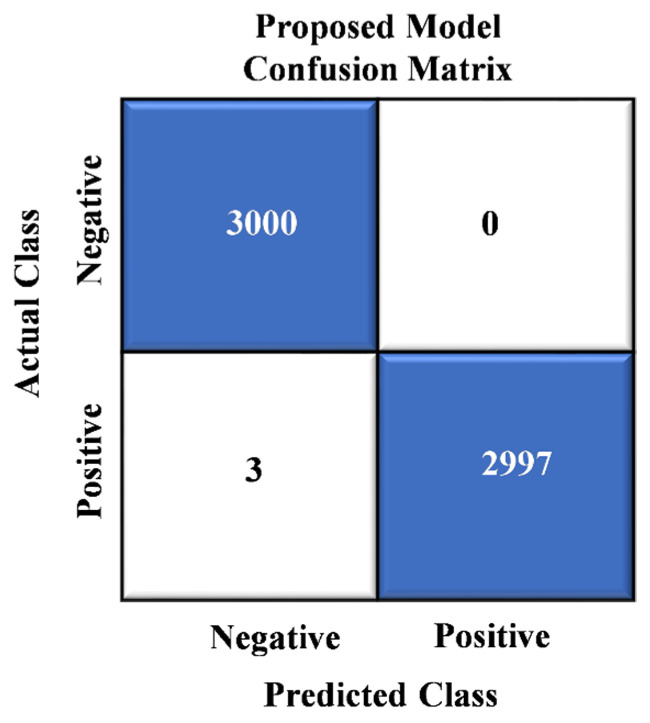
Confusion matrix analysis of proposed model.

In Figure 5, we showcase the results of the confusion matrix analysis for our proposed model. The evaluation of the model on a dataset comprising 6000 images reveals a remarkable level of accuracy, with a mere three instances of misclassification. This impressive performance underscores the model’s ability to categorize most images in the dataset effectively. Notably, these rare misclassifications involve images erroneously labeled as “crack images”.

### 4.5. Performance Comparison with Some Standard TL Models

Table 3 presents a performance comparison of different neural network models in terms of precision, recall, F1 Score, and accuracy for a specific task. The models include ResNet-50, ResNet-152, DenseNet-121, DenseNet-169, DenseNet-201, EfficientNet-B4, EfficientNet-B5, EfficientNet-B6, and a proposed model. Among these models, ResNet-152 and the proposed model exhibit the highest precision, recall, F1 Score, and accuracy, all at around 99.95%. ResNet-50 also performs exceptionally well, with scores slightly lower at 99.91%. The DenseNet models achieve good results in the mid-90s range, with DenseNet-121 being the lowest performer at 96.43%. Finally, the EfficientNet models perform impressively, with scores ranging from 99.76% to 99.93%. The proposed model stands out as it achieves top-tier results, making it a strong candidate for the task, potentially outperforming the base models in precision, recall, and F1 Score. Overall, these models indicate high accuracy and effectiveness in their respective roles, with the choice of the best model depending on specific application requirements and trade-offs.

### 4.6. Performance Comparison with Existing Model

Table 4 shows various reference methods and their corresponding accuracy scores in a research study. Here are the details: Alipour et al. [2]—They utilized “ResNet-18” and obtained an accuracy of 97.95%. Huyan et al. [16]—They employed the “CrackU-Net” method and achieved an accuracy of 99.01%. Liu et al. [25]—Their method, “NB-FCN”, achieved an accuracy of 97.96%. Wan et al. [26]—They employed “DenseNet” and achieved an accuracy of 95.91%. Pu et al. [33]—They used the “Ced-Net” method and achieved an accuracy of 98.90%. Shim et al. [36]—Their approach was the “Multiscale Adversarial NN”, which yielded an accuracy of 98.17%. Yang et al. [43]—They used “FCN” and obtained an accuracy of 97.96%. Song et al. [46]—Their approach was “TDB-Net”, with an accuracy of 98.00%. Proposed Model—The authors introduced an “Ensemble” model, which outperformed the others with an impressive accuracy of 99.95%. The accuracies achieved by various methods in the context of the research study, with the Proposed Model achieving the highest accuracy.

### 4.7. Feature Map Analysis and Grad Can Visualization

Feature map analysis is crucial to understanding the inner workings of CNN architectures. Selected baseline architecture design principles were extracted and represent features from input data.

1. MobileNet

**Figure 6:**
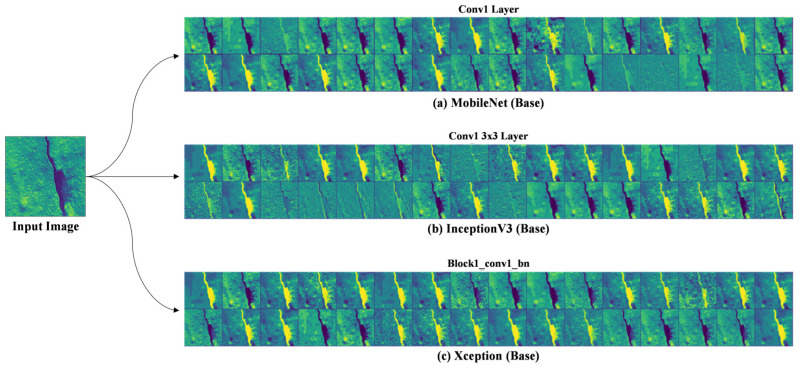
Feature map analysis of baseline models: (**a**) MobileNet, (**b**) InceptionV3, and (**c**) Xception.

MobileNet is designed to be a lightweight and efficient CNN architecture, particularly suitable for mobile and embedded devices. It employs depthwise separable convolutions, which consist of two consecutive layers: depthwise convolution and point-wise convolution. Feature Map Structure: MobileNet’s depthwise separable convolutions result in a compact set of feature maps. The depthwise convolution operates on individual input channels independently, while the point-wise convolution combines the outputs across channels. This leads to fewer parameters and computations, making MobileNet well-suited for resource-constrained environments. Hierarchical Features: MobileNet’s feature maps capture hierarchical information, with earlier layers focusing on low-level features like edges and textures, while deeper layers’ capture more abstract and high-level representations of objects and patterns as shown in Figure 6.

2. InceptionV3

InceptionV3 is known for its inception modules, designed to capture features at multiple scales and levels of abstraction. This architecture promotes improved feature representation and object recognition. Multiple Paths: Inception modules in InceptionV3 utilize multiple convolutional paths with different kernel sizes. This enables the network to capture fine-grained details and global contextual information in parallel. Feature Fusion: Feature maps (Figure 6) from different paths are concatenated or fused, allowing the network to learn diverse features. This results in more affluent and more discriminative feature representations.

3. Xception

Xception is an extension of the Inception architecture that takes the concept of depthwise separable convolutions to the extreme. It replaces standard convolutions entirely with depthwise separable convolutions. Extreme Depth Separation: Xception’s feature map analysis reveals an exceptionally deep network that relies exclusively on depthwise convolutions. This design promotes increased feature diversity and efficiency. Fine-Grained Features: Xception’s depthwise separable convolutions allow it to capture fine-grained details in the feature maps (Figure 6. Feature Map Analysis of Baselines models: (a) MobileNet, (b) InceptionV3, and (c) Xception), making it well-suited for tasks requiring precise localization of objects or features.

Our selected MobileNet, InceptionV3, and Xception are three distinct CNN architectures with varying approaches to feature map analysis in Figure 6. MobileNet prioritizes efficiency, InceptionV3 focuses on capturing diverse features at multiple scales, and Xception pushes the boundaries of depthwise separable convolutions for fine-grained feature extraction. Understanding the nuances of these feature map analyses can help researchers and practitioners choose the right architecture for their specific computer vision tasks.

GRAD-CAM is a powerful technique in the field of computer vision that helps explain the decisions made by deep neural networks, specifically CNNs in image classification tasks. It provides visualizations highlighting the regions within an input image that contribute the most to the network’s classification decision.

MobileNet is known for its lightweight architecture, making it suitable for mobile and embedded devices. When applying GRAD-CAM to MobileNet, the following steps are typically followed:

- Forward Pass. The input image is fed through the MobileNet model to obtain the final classification score.
- Backward Pass. The gradients of the target class score concerning the feature maps of the final convolutional layer are computed.
- Weighted Sum. The gradients are then weighted by the activations in the feature maps to determine which regions have the most influence on the classification decision.
- Heatmap Visualization. Finally, the weighted gradients are used to generate a heatmap highlighting the input image’s critical regions.

InceptionV3 is a more complex CNN architecture with multiple inception modules. When applying GRAD-CAM to InceptionV3, the procedure is similar to MobileNet but involves the following key steps:

- Multiple Branches. InceptionV3 has various branches in its architecture. The gradients and activations are computed for each component and combined to obtain a comprehensive heatmap.
- Hierarchical Analysis. The hierarchical structure of InceptionV3 allows for a more detailed analysis of which parts of the image contribute to different levels of abstraction in the network’s decision-making.

Xception is another advanced CNN architecture that extends the idea of depthwise separable convolutions. Applying GRAD-CAM to Xception involves the following steps:

- Depthwise Separable Layers. Xception’s depthwise separable layers are treated similarly to standard convolutional layers during the GRAD-CAM process.
- High-Level Abstractions. Xception excels at capturing high-level image features, and GRAD-CAM can help identify the specific regions of the image that lead to these high-level abstractions.

**Figure 7:**
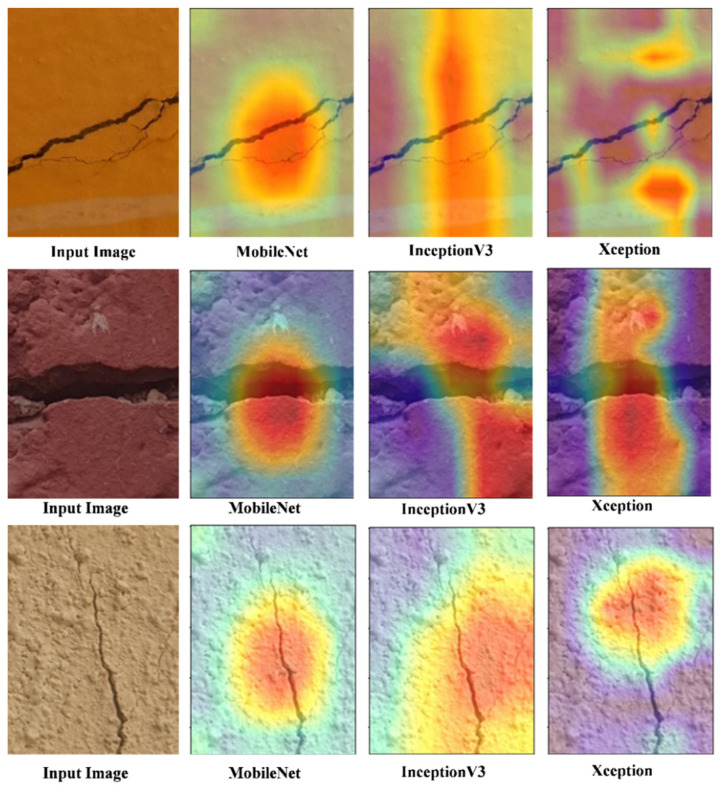
Grad-CAM analysis of baseline models.

As shown in Figure 7, the resulting heatmaps from GRAD-CAM of baselines models provide valuable insights into the neural network’s decision-making process by highlighting which parts of an input image were most influential in making a particular classification decision. This information is crucial for model interpretability, debugging, and understanding the inner workings of these CNN architectures.

### 4.8. Ablation Studies

Table 5 provides a performance comparison of different baseline models, stack ensemble, and proposed models based on several evaluation metrics. These models were likely trained and tested on a road crack dataset, such as the detection of road information for autonomous vehicles. MobileNet (Base), InceptionV3 (Base), and Xception (Base) are three baseline models, each achieving high levels of precision, recall, F1 Score, and accuracy, around 96% to 98%. These metrics reflect their ability to classify and recognize crack, and non-crack images in the dataset correctly they were tested on. The stack ensemble model outperforms the baseline models, with precision, recall, F1 Score, and accuracy all improved to around 98.8%. the stack ensemble model combines the predictions of selected baseline models to boost overall performance. The proposed model stands out as the top performer, achieving exceptionally high scores in all metrics, with precision, recall, F1 Score, and accuracy around 99.95%. This suggests that the proposed model has achieved remarkable accuracy in classifying objects or data points, making it the best-performing model among the ones listed.

**Figure 8:**
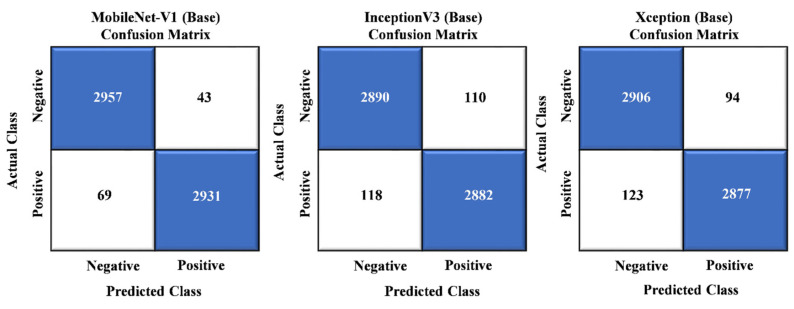
Confusion matrix analysis of baseline models.

Figure 8 provides a detailed breakdown of the misclassifications of the three independent base models: MobileNet, InceptionV3, and Xception. Among the 6000 evaluated instances, MobileNet had 112 misclassifications, InceptionV3 had 228, and Xception had 217. These statistics reveal the individual performance of each base model. However, it is worth highlighting that our suggested model, which combines the strengths of these base models, exhibited a significantly lower error ratio than each. This outcome demonstrates our proposed model’s enhanced predictive accuracy and superior performance, making it a more effective choice for the task at hand.

**Figure 9:**
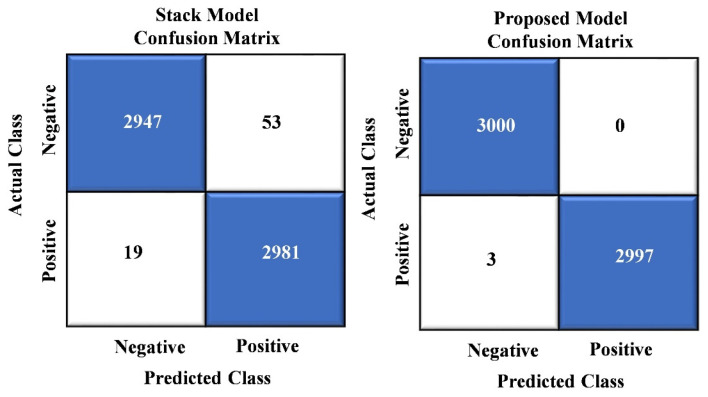
Confusion matrix visualization between stack and proposed model.

Figure 9 presents a comprehensive analysis of misclassifications, comparing the stack ensemble technique with our proposed model. In a set of 6000 instances, the stack ensemble model exhibited 72 misclassifications, while the proposed model showed a meager count of just three incorrect classifications. These results outperform the baseline models shown in Figure 8 and surpass the stack ensemble technique. Our proposed model achieved a substantially lower error rate, solidifying its position as a superior choice due to its enhanced predictive accuracy and outstanding performance.

## 5. Discussion

This paper proposed a novel hybrid approach for road information detection in autonomous vehicles, utilizing both positive and negative images. The proposed method combines a Random Forest classifier with transfer learning techniques. Training a CNN is often challenging due to limited labeled data availability in the crack and non-crack imaging scenarios. To address this challenge, our study leverages layers from three pre-trained models, Xception, InceptionV3, and MobileNet. This fusion of pre-trained models with Random Forest prevents overfitting and enhances high-dimensional feature extraction for improved performance. Moreover, we employ data augmentation to enhance the model’s generalization capacity and accuracy.

Integrating these stacked features with Random Forest yields significant improvements in accuracy, precision, recall, and F1 Score compared to alternative approaches explored in our research. Detailed results are presented in Table 2 and Table 4 and Figure 8 and Figure 9. Our suggested model consistently achieves the highest classification accuracy through extensive experimentation. Table 3 and Table 5 and Figure 5 further underscore the superior performance of our proposed model, which harnesses the combined strengths of multiple models for enhanced road information detection.

The practical implementation of our proposed model is that road crack detection for autonomous vehicles using deep learning is trained on vast datasets of road images to accurately identify and localize cracks. The proposed model is integrated into the vehicle’s sensor suite, enabling real-time detection and response to potential road hazards, ensuring safer navigation.

## 6. Strength and Limitation

The paper addresses the critical task of road crack detection, particularly essential for inspecting and evaluating the integrity of concrete pavement structures, such as in autonomous vehicle road detection scenarios. Traditional image-based methods often struggle with complex preprocessing requirements to extract crack features, making them less practical for real-world scenarios characterized by various forms of noise on concrete surfaces. To overcome these challenges, the research proposes an innovative approach that combines a Random Forest machine learning classifier with a DCNN (deep convolutional neural network). This hybrid model effectively leverages the strengths of both techniques, enhancing crack detection performance significantly. Through rigorous experimentation, the paper identifies an optimal base learning rate of 0.001, crucial for model training, and achieves an impressive maximum validation accuracy of 99.97%, contributing significantly to the overall strength of the approach.

The current study could be enhanced by further integrating real-time data streams and adaptive learning algorithms. This integration could lead to improved model performance and scalability. Real-time data streams provide up-to-date information, allowing the model to make more accurate predictions or decisions based on the latest data. Additionally, adaptive learning algorithms can adjust their behavior based on new data or changing circumstances, leading to more flexible and efficient models. While we aim to ensure the generalization of our proposed method by working with other datasets, future research will focus on further validating its applicability across diverse data sources and real-world scenarios.

## 7. Conclusions

Road crack detection is a critical and challenging task, a fundamental aspect of infrastructure maintenance and autonomous vehicle technologies. The findings of this study contribute significantly to the field of computer vision and image-based defect detection. Several key takeaways and contributions emerge from this research. First and foremost, this study offers a practical solution to the road crack detection problem by leveraging a hybrid approach. Combining a Random Forest machine learning classifier with state-of-the-art deep convolutional neural network (CNN) models, namely MobileNet, InceptionV3, and Xception, yields a robust and adaptable system. This hybrid approach effectively mitigates the complexities of preprocessing and noise in real-world concrete surfaces. Moreover, through systematic experimentation, the research identifies an optimal base learning rate of 0.001, achieving an exceptional maximum validation accuracy of 99.97%. This discovery is pivotal in enhancing model training and overall detection performance. The comprehensive testing phase, conducted on a separate dataset of 6000 photos with 224 × 224-pixel resolutions, underscores the robustness and generalization capabilities of the proposed technique. The ability to accurately identify road fractures in images captured on actual concrete surfaces demonstrates the practical applicability of the approach in real-world scenarios. These contributions collectively underscore the significance of this research in the domain of defect detection and infrastructure maintenance. The hybrid approach, optimal learning rate determination, and thorough testing advance the state of the art in road crack detection and hold promise for broader applications in computer vision and machine learning.

As we move forward, further research may explore the integration of real-time monitoring systems, the potential for scalability, and the extension of this approach to address additional infrastructure maintenance challenges. In doing so, this work lays a strong foundation for improving the safety and reliability of our transportation systems. It contributes to the advancement of smart cities and autonomous vehicle technologies.

## Figures and Tables

**Table 1:** Dataset distribution with sample of each class.

Example	Class	Train	Test	Total
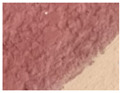	Negative	12,000	3000	15,000
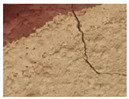	Positive	12,000	3000	15,000
Total		24,000	6000	30,000

**Table 2:** Classification performance.

Models	Precision	Recall	F1 Score	Accuracy	Test Time
MobileNet (Base)	98.13	98.13	98.13	98.13	0:00:13.92
InceptionV3 (Base)	96.38	96.38	96.38	96.38	0:00:15.20
Xception (Base)	96.20	96.20	96.19	96.20	0:00:13.81
Proposed Model	99.95	99.94	99.94	99.95	0:00:19.86

**Table 3:** Performance comparison with some standard pre-trained model.

Models	Precision	Recall	F1 Score	Accuracy
ResNet-50 (Base)	99.91	99.91	99.91	99.91
ResNet-152 (Base)	99.95	99.95	99.94	99.95
DenseNet-121 (Base)	96.45	96.43	96.43	96.43
DenseNet-169 (Base)	97.18	97.18	97.18	97.18
DenseNet-201 (Base)	97.83	97.81	97.81	97.81
EfficientNet-B4 (Base)	99.85	99.84	99.84	99.85
EfficientNet-B5 (Base)	99.76	99.76	99.76	99.76
EfficientNet-B6 (Base)	99.93	99.93	99.93	99.93
Proposed Model	99.95	99.94	99.94	99.95

**Table 4:** Performance comparison with existing models.

Reference	Method	Precision	Accuracy
Alipour et al. [2]	ResNet-18	-	97.95%
Huyan et al. [16]	CrackU-Net	98.56%	99.01%
Liu et al. [25]	NB-FCN	81.73%	97.96%
Wan et al. [26]	DenseNet	-	95.91%
Pu et al. [33]	Ced-Net	93.58%	98.90%
Shim et al. [36]	Multiscale Adversarial NN	-	98.17%
Yang et al. [43]	FCN	83.30%	97.96%
Song et al. [46]	TDB-Net	-	98.00%
Proposed Model	Ensemble	99.95%	99.95%

**Table 5:** Performance analysis between proposed model and others.

Models	Precision	Recall	F1 Score	Accuracy
MobileNet (Base)	98.13	98.13	98.13	98.13
InceptionV3 (Base)	96.38	96.38	96.38	96.38
Xception (Base)	96.20	96.20	96.19	96.20
Stack Ensemble	98.80	98.80	98.79	98.80
Proposed Model	99.95	99.94	99.94	99.95

## Data Availability

Dataset available on request from the authors.
